# Endoscopic surgical treatment for rhinogenic contact point headache: systematic review and meta-analysis

**DOI:** 10.1007/s00405-021-06724-6

**Published:** 2021-03-06

**Authors:** Antonino Maniaci, Federico Merlino, Salvatore Cocuzza, Giannicola Iannella, Claudio Vicini, Giovanni Cammaroto, Jérome R. Lechien, Christian Calvo-Henriquez, Ignazio La Mantia

**Affiliations:** 1 Department of Medical and Surgical Sciences and Advanced Technologies “GF Ingrassia”, ENT Section, ENT Department of University of Catania, Via Santa Sofia, 95100 Catania, Italy; 2 Department of ‘Organi Di Senso’, University “Sapienza”, Rome, Italy; 3 Department of Head-Neck Surgery, Otolaryngology, Head-Neck, and Oral Surgery Unit, Morgagni Pierantoni Hospital, Forlì, Italy; 4 Research Committee of the Young Otolaryngologists, International Federations of ORL Societies, Paris, France; 5 Department of Human Anatomy and Experimental Oncology, School of Medicine, UMONS Research Institute for Health Sciences and Technology, University of Mons, Mons, Belgium; 6 Department of Otorhinolaryngology-Head and Neck Surgery, CHU Saint-Pierre, School of Medicine, Université Libre de Bruxelles, Brussels, Belgium; 7 Department of Otolaryngology-Head and Neck Surgery, Foch Hospital, (University of Paris-Saclay), Paris, France; 8 Task Force COVID-19 of the Young-Otolaryngologists of the International Federations of Oto-Rhino-Laryngological Societies (YO-IFOS), Department of Otolaryngology, Hospital Complex of Santiago de Compostela, Santiago de Compostela, Spain

**Keywords:** Rhinogenic contact point headache, Concha bullosa, Septal spur, Septal deviation, Endoscopic surgery

## Abstract

**Purpose:**

This meta-analysis study was designed to analyze endoscopic surgery’s role in treating rhinogenic contact point headache.

**Methods:**

We performed a comprehensive review of the last 20 years’ English language regarding Rhinogenic contact point headache and endoscopic surgery. We included the analysis papers reporting post-operative outcomes through the Visual Analogue Scale or the Migraine Disability Assessment scale.

**Results:**

We provided 18 articles for a total of 978 RCPH patients. While 777 (81.1%) subjects underwent functional nasal surgery for RCPH, 201 patients (20.9%) were medically treated. A significant decrease from the VAS score of 7.3 ± 1.5 to 2.7 ± 1.8 was recorded (*p* < 0.0001). At quantitative analysis on 660 patients (11 papers), surgical treatment demonstrated significantly better post-operative scores than medical (*p* < 0.0001).

**Conclusion:**

At comparison, surgical treatment in patients with rhinogenic contact points exhibited significantly better values at short-term, medium-term, and long term follow up. Endoscopic surgery should be proposed as the choice method in approaching the symptomatic patient.

## Introduction

The International Headache Society distinguishes headache disorders between symptomatic primary or idiopathic, secondary headache and orofacial pain disorders including neuralgia and nasosinusal causes of headache [1–3].

Already in 1943, McAuliffe et al. explicated that the stimulation of specific anatomical structures of the nasal cavities could lead to trigeminal nerve stimulation and the release of substance P with referred headache in the absence of nasosinusal inflammatory disorders [4, 5].

Later, Zechner et al. defined the rhinogenic contact point headache (RCPH) as the headache symptomatology associated with contact between the lateral wall mucosa to the nasal septum [6].

In 2004 the Headache Classification Subcommittee of the International Headache Society included rhinogenic contact point headache (RCPH) among the secondary nasosinusal causes of headache [1].

RCPH is distinguished by several possible anatomical abnormalities such as septal spurs or middle turbinate disorders such as hypertrophic, deformed or hyperpneumatized (concha bullosa), in the absence of inflammation of nasal mucosa. RCHP is quickly detectable and quantified by sinonasal endoscopy or computed tomography [7–11].

As emerged in the literature, RCPH is a controversial clinical entity [12, 13]. Different authors analyzed the endoscopic nasal surgery effect as a possible therapeutic strategy to treat cases of suspected rhinogenic headaches associated with RCPH [13–16]. Validated subjective questionnaires, such as the Visual Analog Scale (VAS) and Migraine Disability Assessment (MIDAS), were commonly used in the literature to estimate the outcomes of reduction headache symptoms in post-surgery [17–24].

Cantone et al. in 2014 reported better outcomes in 53 patients treated with endoscopic surgery for rhinogenic headache [25]. Patients with initial grade III and IV on MIDAS scores at 3 and 6 months of follow-up switched grades I and II or presented total symptoms resolution. Guyuron et al., in a five-year outcome retrospective study, stated the significant improvement (*p* < 0.0001) of all scores analyzed (26). In contrast, Bieger-Farhan et al. although it found a contact point in 55% of patients analyzed with routine coronal paranasal sinus CT, it found a significant association with nasal obstruction and smell reduction (*p* < 0.01) but not with facial pain [27].

According to this evidence, other authors have hypothesized that, in patients undergoing surgery, the benefit of referred symptoms is related to the placebo effect [28–34].

The cognitive dissonance phenomenon and the consequent subjective perception reduction would be responsible for the temporary symptom reduction within two years of the intervention (short–medium term) [28–30].

To our knowledge, no meta-analysis studied the outcomes of nasal surgery in rhinogenic headaches with RCHP, confirming/denying the evidence of the isolated studies. In this paper, we performed a systematic review and a meta-analysis to evaluate nasal surgery’s role in improving symptoms of rhinogenic headaches with RCHP.

## Materials and methods

### Protocol data extraction and outcomes evaluated

The authors A.M and F.M analyzed the data from the literature. A discussion solved any disagreements among the study team members. Included studies were thus analyzed to obtain all available data and guarantee eligibility for all subjects. Patient’s characteristics, symptoms, diagnostic procedures, treatment modalities, outcomes scores (VAS and MIAS), and follow-up were collected.

The effect of surgical treatment on rhinogenic headaches with RCHP has been evaluated comparing Pre- and post-operative VAS and MIDAS scores; subsequently, surgical and medical therapy outcomes were also compared.

### Electronic database search

According to the PRISMA checklist for review and meta-analysis, we performed a systematic review of the current literature.

PubMed, Scopus and Web of Science electronic databases were searched for studies on rhinogenic contact point headache/rhinogenic headaches of the last 20 years literature (from December 1st 2000 to December 1st 2020) by two different authors. The related search keywords were used: “Rhinogenic Headache”, “Contact Point Headache”, “nasal endoscopy headache”, “nasal surgery headache”, and “nasal headache”. The “Related articles” option on the PubMed homepage was also considered. The investigators examined titles and abstracts of papers available in the English language. The identified full texts were screened for original data, and the related references were retrieved and checked manually for other relevant studies.

### Inclusion and exclusion criteria

Studies were included when the following criteria were met:

1. Original articles;
2. We excluded to the study inclusion case report, editorial, letter to the editor, or review;
3. The article was published in English;
4. The studies included only clinically confirmed cases of rhinogenic point of contact headache;
5. The studies reported detailed information on pre-operative subjective evaluation through a validated questionnaire such as the Visual analogue scale (VAS) or the Migraine Disability Assessment Test (MIDAS) or radiological scores obtained after CT analysis;
6. The studies mentioned detailed information about post-operative treatment outcomes;

### Statistical analysis

This protocol was performed in line with the approved reporting items’ quality requirements for systematic review and meta-analysis protocols (PRISMA) declaration [35]. Moreover, the studies’ quality assessment (QUADAS-2) instrument was adopted to estimate the included studies’ study design features [36].

Statistical analysis was performed using statistical software (IBM SPSS Statistics for Windows, IBM Corp. Released 2017, Version 25.0. Armonk, NY: IBM Corp). Furthermore, we used random-effects modelling (standard error estimate = inverse of the sample size) to estimate the summary effect measures by 95% confidence intervals (CI), and subsequent forest plots were generated through the Review Manager Software (REVMAN) version 5.4 (Copenhagen: The Nordic Cochrane Centre: The Cochrane Collaboration). We calculated the inconsistency (*I*2 statistic) and established the values for low inconsistency = 25%, moderate inconsistency = 50%, and high inconsistency = 75% [37].

## Results

### Retrieving researches

**Fig. 1:**
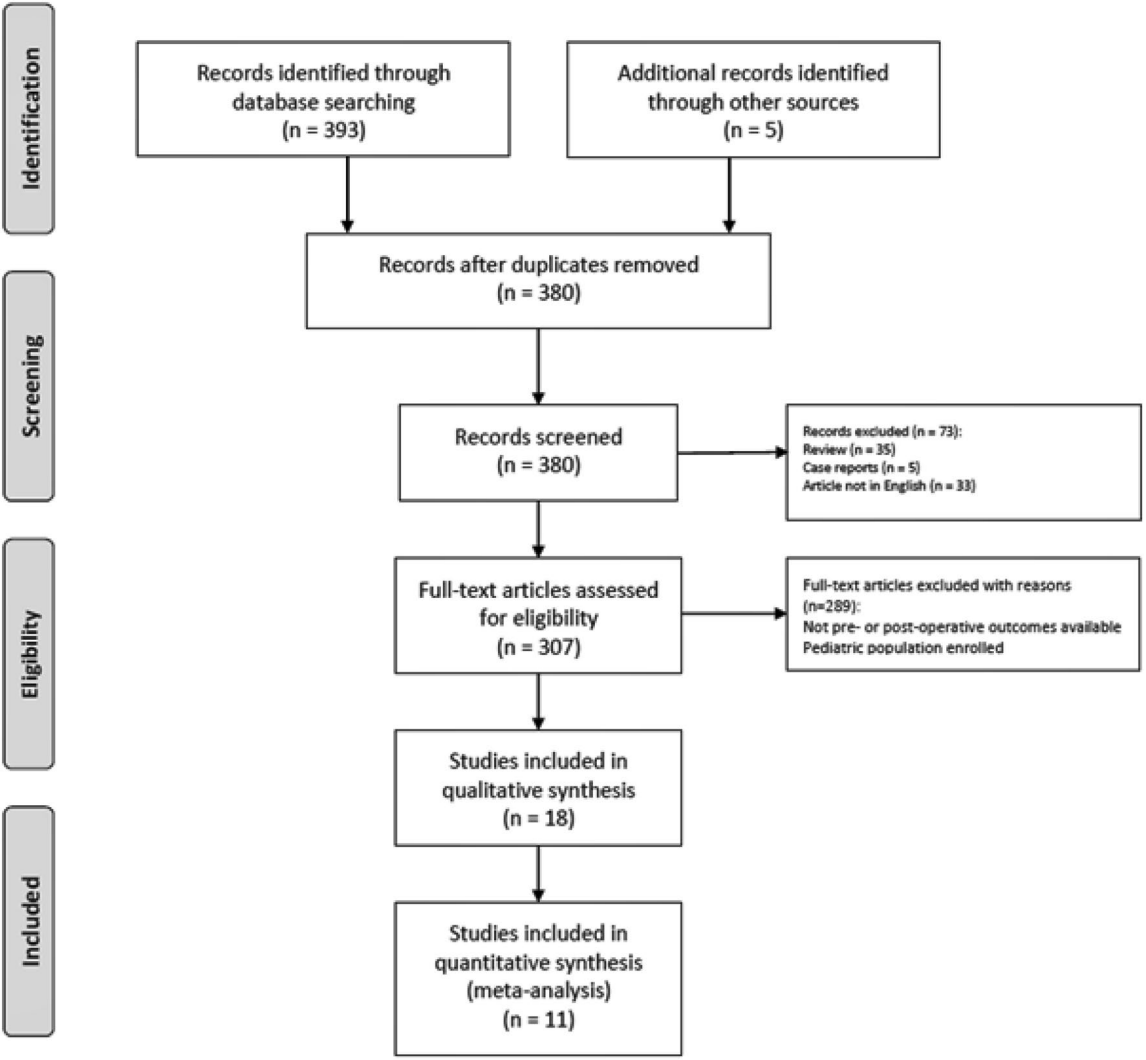
PRISMA flow diagram

**Fig. 2:**
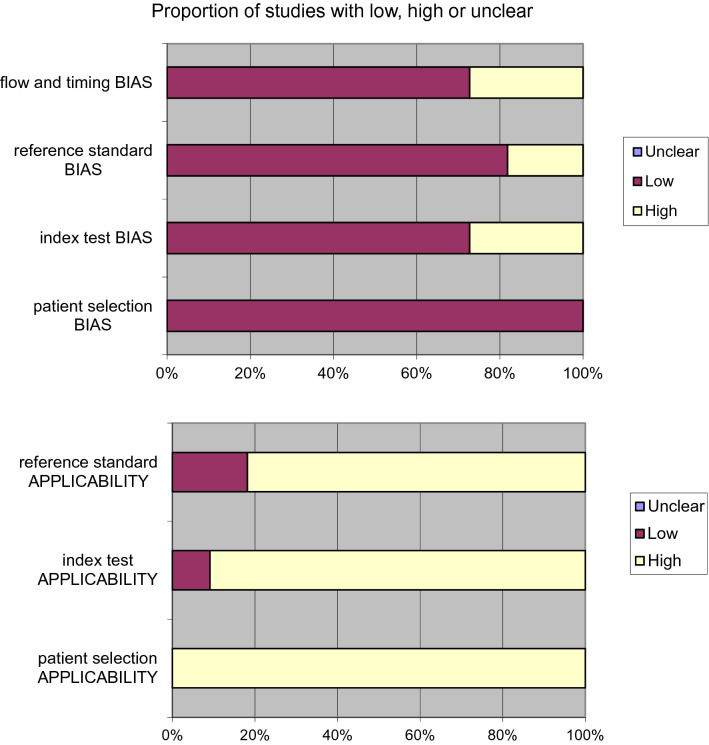
QUADAS-2: the graphical display shows the possible risk of bias

The systematic review of the literature identified 398 potentially relevant studies (Fig. 1). After removing the duplicates and applying the criteria listed above, an overall number of 380 records screened were potentially relevant to the topic. Through the records analysis and subsequent articles full-text screening, we excluded all the studies that did not match inclusion criteria (*n* = 362). The remaining 18 papers were included in qualitative synthesis papers for the data extraction. Moreover, due to the meta-analysis established criteria, we excluded seven papers (absence of data) and considered 11 studies for quantitative analysis. A graphical display of QUADAS-2 results is shown in Fig. 2 summarized the possible risk of bias.

### Patients features and surgery

We provided 18 articles in our systematic literature review for a total of 978 RCPH patients. The patients’ average age was 36.81 ± 16 years. The average follow-up of the study was 37.05 ± 38.53 ranging from 1 to 127 months.

The major sinonasal disorders associated with rhinogenic headache were septal spur combined with concha bullosa in 757 (79%) patients, while isolated septal spur or chonca bullosa in 99 (10.3%) and 102 (10.7%) cases, respectively.

Of these, 777 (81.1%) subjects underwent functional nasal surgery for RCPH; whereas, 201 patients (20.9%) were treated with medical therapy (Table 1). All patients treated with surgery have previously reported failure of medical therapy.

**Table 1:** A Lidocaine test was performed prior treatment

References/year	Study design	Subjective assessment	Treatment features	Surgical approach	Follow-up	Post-operative outcomes
Abu-Samra et al. 2011	Prospective controlled single-blinded study	VAS	Septal spur and/or chonca bullosa in 42 subjects	Standard and/or endoscopic septoplasty and/or turbinateplasty	1–48 months	VAS ↓ improved in 81% of surgical patients (p = 0.001)
Altin et al. 2019	Retrospective non-randomized controlled study	VAS	Septal spur in 51/99 surgical subjects vs 48/99 medical ones	Endoscopic septoplasty	1–6 months	VAS ↓ improved of 79.8% in surgical patients vs ↓ 7% in medical ones (*p* = 0.01)
Behin et al. 2004	Retrospective uncontrolled study	VAS	Septal spur and/or chonca bbullosa in 21/50 subjects	Standard and/or endoscopic septoplasty and/or turbinateplasty	1–62 months	VAS ↓ improved in 95.8% of patients till no headache in 42.9% (*p* < 0.001)
Bektas et al. 2011	Retrospective uncontrolled study	VAS	Septal spur and/or chonca bullosa in 36 subjects	Endoscopic septoplasty	1–6 months	VAS ↓ improved in 100% of patients till no headache in 52.7% (*p* < 0.001)
Bilal et al. 2013	Prospective uncontrolled study	VAS	Septal spur and/or chonca bullosa in 65 subjects	Standard and/or endoscopic septoplasty and/or turbinateplasty	1–12 months	VAS ↓ improved in 52% of patients till no headache in 12.3% (p < 0.001)
Cantone et al. 2014	Retrospective randomized controlled study	VAS/ MIDAS/ Lund-Mackay	Chonca bullosa 53/102 in subjects vs 49/102 medical ones	Endoscopic chonca plasty	1–6 months	VAS ↓ improved in 81% of patients while MIDAS ↓ 100% GRADE 3–4 switched to lower classes till no headache in 44% (*p* < 0.05)
Guyuron et al. 2011	Retrospective randomized controlled study	VAS/MIDAS/ MOS SF-36/ MSQoL	Septal spur and/or chonca bullosa in 79 subjects	Standard and/or endoscopic septoplasty and/or turbinateplasty	1–60 months	All Scores ↓ improved in 90% of patients till no headache in 28% (*p* < 0.001 in all cases)
Huang et al. 2008	Retrospective uncontrolled study	VAS	Septal spur and/or chonca bullosa in 66 subjects	Standard and/or endoscopic septoplasty and/or turbinateplasty	1–127 months	VAS ↓ improved in 81.8% of patients (*p* < 0.001)
Hye Wee et al. 2015	Prospective uncontrolled study	VAS/Lund-Mackay	Septal spur and/or chonca bullosa in 41/356 subjects	Standard and/or endoscopic septoplasty and/or turbinateplasty	1–16 months	VAS ↓ improved in 80% of patients till no headache in 58.5% (*p* < 0.05)
Kunachak et al. 2002	Prospective uncontrolled study	VAS	Septal spur and/or chonca bullosa in 55 subjects	Endoscopic middle turbinate lateralization	1–84 months	VAS ↓ improved in all cases 100% till no headache in 87% (*p* < 0.001)
La Mantia et al. 2017	Retrospective randomized controlled study	VAS/MIDAS	Septal spur and/or chonca bullosa in 47/94 surgical subjects vs 47/94 medical ones	Standard and/or endoscopic septoplasty and/or turbinateplasty	1–6 months	VAS ↓ and MIDAS ↓ improved in 68% of surgical patients vs in 36% of medical ones (*p* < 0.001 in both scores)
Madani et al. 2013	Prospective uncontrolled study	VAS	Septal spur and/or chonca bullosa in 30 in subjects	Endoscopic septoplasty	1–6 months	VAS ↓ improved of 72% in surgical patiens (p = 0.013)
Mariotti et al. 2009	Prospective uncontrolled study	VAS/ Lund-Mackay	Septal spur and/or chonca bullosa in 33 subjects	Standard and/or endoscopic septoplasty and/or turbinateplasty	1–24 months	VAS ↓ improved in 84.8% of patients (*p* < 0.01)
Mohebbi et al. 2009	Prospective non-randomized study	VAS	Septal spur and/or chonca bullosa in 36 subjects	Standard and/or endoscopic septoplasty and/or turbinateplasty	1–48 months	VAS ↓ improved in 83% of patients till no headache in 11% (*p* = 0.05)
Peric et al. 2016	Retrospective uncontrolled study	VAS	Septal spur and/or chonca bullosa in 42 subjects	Standard and/or endoscopic septoplasty and/or turbinateplasty	1–24 months	VAS ↓ improved in 88.1% of patients
Welge-Luessen et al. 2003	Prospective uncontrolled study	VAS	Septal spur and/or chonca bullosa in 20 subjects	Standard and/or endoscopic septoplasty and/or turbinateplasty	1–120 months	VAS ↓ improved in 75% of patients till no headache in 30% (*p* = 0.018)
Yarmohammadi et al. 2014	Prospective randomized controlled study	VAS	Septal spur and/or chonca bullosa in 22/44 surgical subjects and 22/44 medical ones	Standard and/or endoscopic septoplasty and/or turbinateplasty	1–6 months	VAS ↓ better improvements in surgical group than medical (scores 0 vs 5.5 respectively; *p* < 0.001)
Yazici et al. 2010	Retrospective randomized controlled study	VAS	Septal spur and/or chonca bullosa in 38/53 surgical subjects vs 15/53 medical ones	Standard and/or endoscopic septoplasty and/or turbinateplasty	1–6 months	VAS ↓ improved of 61% in surgical patients vs 4.5% in medical ones

*VAS* Visual Analogue Scale, *MIDAS* Migraine Disability Assessment, *MOS SF-36* Medical Outcomes Study 36-Item Short-Form, *MSQoL* migraine-specific quality of life

### VAS outcomes comparison in surgical patients

**Fig. 3:**
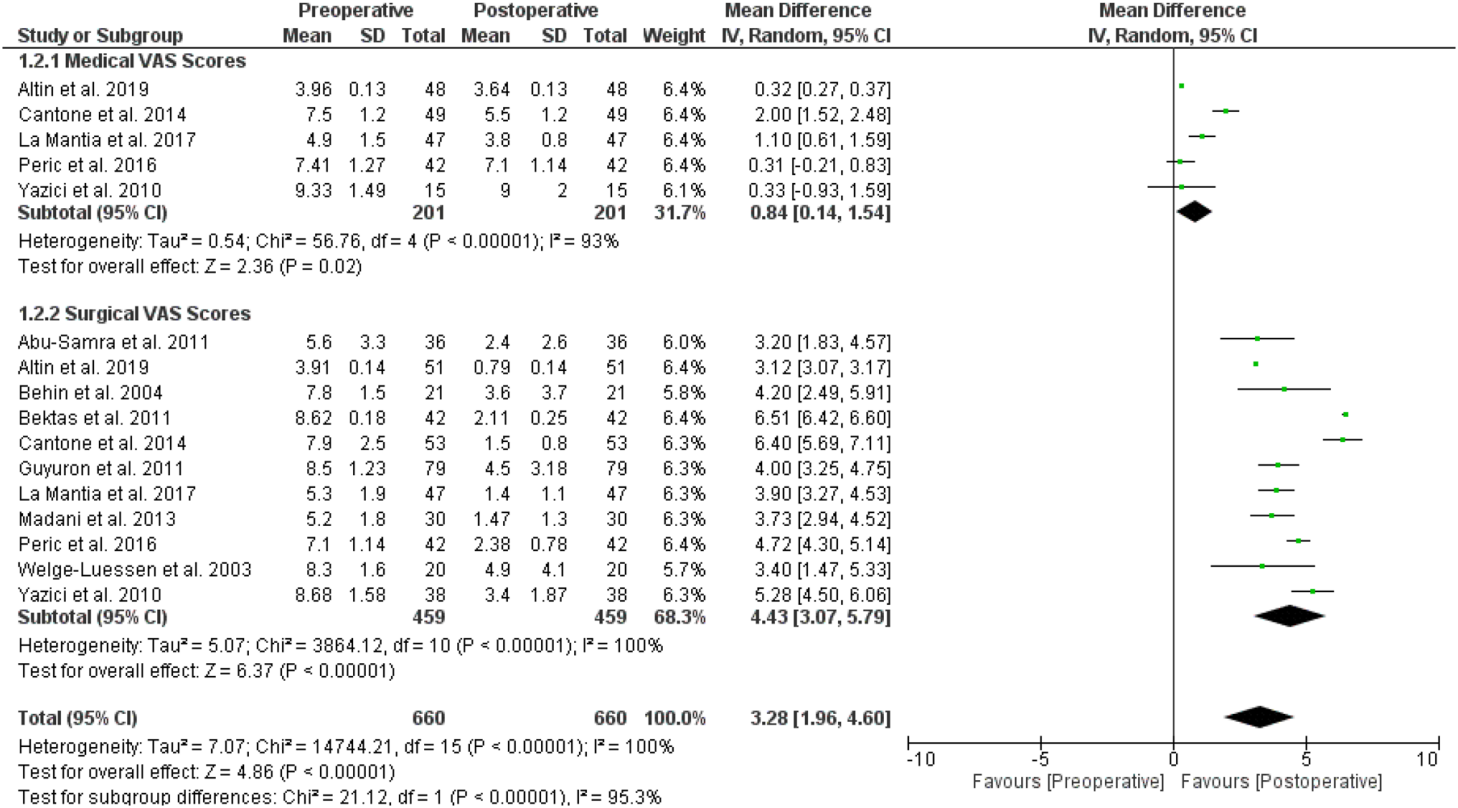
Forest plot between surgical vs medical pre-operative and post-operative VAS scores. *CI* confidence interval, *SD* standard deviation

Of the studies included, 11/18 papers (459 patients) reported both pre-and post-operative mean value ± SD of the VAS scores (Fig. 3). In particular, a significant VAS score reduction from the value of 7.3 ± 1.5 to 2.7 ± 1.8 was estimated (*p* < 0.001).

The analysis using random-effects modeling for 459 patients demonstrated a MD of 4.43 [95% CI 3.07, 5.79] of the VAS score, overall effect *Z *score = 6.37, *Q* statistic *p* < 0.00001 (statistically significant heterogeneity), *I*2 = 100% (high inconsistency) as described in Fig. 3.

### Short–medium vs long-term outcomes

**Fig. 4:**
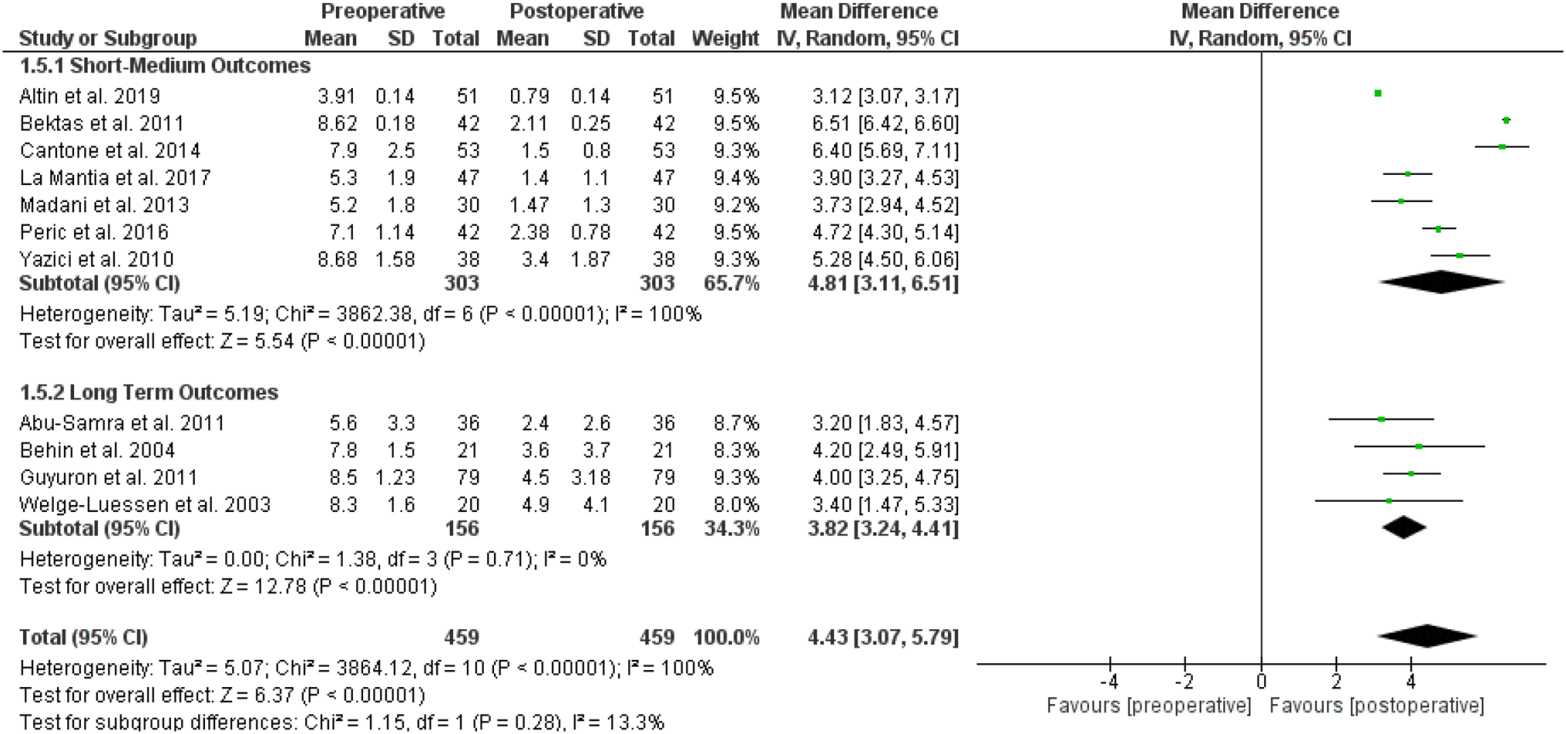
Subanalysis forest chart distinguishing patients based on follow-up term. *CI* confidence interval, *SD* standard deviation

Sub-analysis of postoperative results stratified by short–medium vs long-term follow-up are shown in Fig. 4. The short–medium term group (1–24 months) of 303 patients presented at random-effects modeling a score MD of 4.81 [95% CI 3.11, 6.51], overall effect *Z* score = 5.54 (*p* < 0.00001), Q statistic *p* < 0.00001 (statistically significant heterogeneity), *I*2 = 100% (high inconsistency). On the other hand, the long-term group (25–120 months) of 156 patients reported a score MD of 3.82 [95% CI 3.24, 4.41], overall effect *Z* score = 12.78 (*p* < 0.00001), *Q* statistic *p* = 0.71 (no statistical heterogeneity), *I*2 = 0% (no inconsistency).

Thus, the test for subgroup differences was not statistically significant (*p* = 0.28, *I*2 = 13.3%).

### Surgical vs medical treatment

Among the selected studies, 11/18 papers compared changes in VAS scores in a total of 459 surgical patients versus 201 undergoing medical treatment (Fig. 3). The topical therapy mainly used was fluticasone propionate nasal spray, every morning in cycles of 15 consecutive days per month up to 6 months of treatment.

**Fig. 5:**
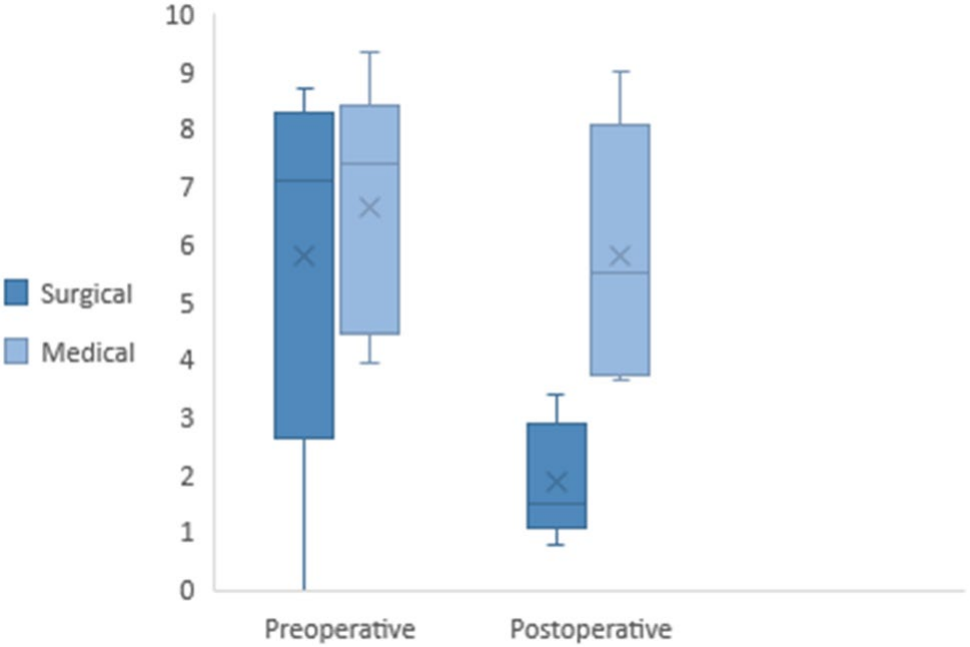
Box plot pre- and post-operative outcomes’ comparison between surgery and medical therapy. Improvement in VAS scores of the medical therapy did not reach statistical significance (*p* = 0.57)

Although both treatments reported a statistically significant reduction in post-operative scores in both groups (*p* < 0.0001 both), the surgical treatment demonstrated significantly better post-operative scores (*p* < 0.0001) (Fig. 5).

Furthermore, medical treatment at random-effects modeling for 201 patients reported a MD of 0.84 [95% CI 0.14, 1.54] VAS score, overall effect *Z* score = 2.36 (*p* = 0.02), *Q* statistic *p* < 0.00001 (statistically significant heterogeneity), *I*2 = 93% (high inconsistency).

Thus, the test for subgroups was statistically significant (p < 0.00001, I2 = 95.3%).

### MIDAS outcomes

Changes in mean MIDAS scores were identified for 120 patients enrolled in three studies (Table 2). Significant improvements occurred after surgical treatment with a reduction from 88 (73%) to 6 (5%) patients for GRADE 3–4 and a corresponding increase in milder symptoms from GRADE 1–2 in 32 (27%) patients at 91 (76%) (*p* < 0.001).

**Table 2:** MIDAS outcomes comparison expresses better study at follow-up after a surgical approach

References	Patients	Pre-operative MIDAS		Postoperative MIDAS			
		Grade 3–4	Grade 1–2	Grade 3–4	Grade 1–2	Grade 0	*p* value
Cantone et al. 2014	53	38 (72%)	15 (28%)	0	30 (56%)	23 (44%)	< 0.00001
La Mantia et al. 2017	47	36 (76.60%)	11 (23.4%)	4 (8.5%)	43 (91.5%)	–	< 0.00001
Segana et al. 2016	20	14 (70%)	6 (30%)	2 (10%)	18 (15%)	–	= 0.0001
Total	120	88 (73%)	32 (27%)	6 (5%)	91 (76%)	–	< 0.00001

The chi-squared statistic reported for all score a *p* value is < 0.00001

Besides, the remaining 23 patients (19.16%) had complete resolution of symptoms at follow-up.

## Discussion

Rhinogenic contact point headache is characterized by a contact between different anatomical structures such as the nasal septum and the middle, superior turbinate or the anteromedial wall of the ethmoid sinus associated with frontal–orbital pain radiating to the root of the nose [15, 16, 26].

RCPH patients frequently come to surgical treatment after years of failure to medical therapy and multiple specialist assessments [17, 21].

In this regard, Peric et al. in 2016 found an overall VAS improvement at 24 months from 7.10 ± 1.14 to 2.38 ± 0.78 (*p* = 0.001), especially in patients with concha bullosa and septal spur (*p* < 0.0001 [33].

Several authors also investigated medical therapy’s role in resolving painful symptoms, often demonstrating unpromising results unlike surgery [10, 18, 25].

Our meta-analysis between 459 undergoing surgical treatment and 201 undergoing medical one clarified the primary role of endoscopic surgery in RCPH patients, reporting an overall surgical success usually reported around 80% (*p* < 0.00001; *Z* = 4.86; *I*2 = 95.3%) (Fig. 3).

In contrast, at the post-operative medical follow-up, no significant better improvement was obtained (*p* = 0.53) (Fig. 5).

However, we identified a risk of bias among the included studies due to the lack of symmetry between patients enrolled in surgical therapy and medical as control. Not all authors included sufficient patients to compare the different treatment modalities or further randomized them into two distinct groups to test the approaches’ differences. Furthermore, selection bias frequently involves many studies in the literature. A rigorous evaluation of possible comorbidities such as allergic rhinitis or differential diagnosis with other causes of headaches is often not performed. In this regard, although the lidocaine test represents the gold standard in RCPH diagnosis, not all authors in the literature perform it before surgical treatment.

Another critical point frequently discussed in the literature is preserving long-term treatment results [28, 38–41].

In a retrospective chart review on 973 patients, West et al. hypothesized that surgery could trigger neuroplasticity processes such as the cognitive dissonance, improving the associated symptoms only temporarily and in a minority of patients [28].

Instead, Welge-Luessen et al. reported in a 10-year longitudinal study data significantly opposite to previously stated [34]. The authors described excellent results in surgical patients with a mean follow-up of 112 months, reporting an overall improvement of up to 65%.

Our meta-analysis, subdividing patients according to average follow-up, confirmed that surgical therapy could lead to optimal results both in the short–medium long-term, with no statistical differences between subgroups (*p* = 0.28) (Fig. 4).

However, almost all studies include not differing RCPH modalities of interventions and the specific anatomical structures responsible, not permitting to distinguish the corresponding results at follow-up through the sub-analysis.

Even in the studies in which long-term follow-up and promising outcomes were reported in both medical and mostly surgical treatment, it was not possible to identify the anatomical structures with the most favorable response to medical or surgical treatment or both.

A further valid tool in evaluating the patient’s symptomatological characteristics with RCPH is represented by the Migraine Disability Assessment Scale (MIDAS) [10, 42].

The systematic literature review found that the comparison between the MIDAS score in patients undergoing surgery led to substantial improvements in the post-operative group. In particular, patients presented an overall Grade 3–4 switch from 73 to 5% while a full resolution was registered in 19% of cases (*p* < 0.001 in all grades).

Several studies analyzed do not have a prospective study protocol nor adequate randomization. Besides, in a few cases, the authors included in the analysis of a control group. It was possible to compare traditional medical therapy’s effects in dealing with headache symptoms.

The initial diagnostic classification was not carried out routinely in all the studies to obtain a diagnostic confirmation of the rhinogenic headache and achieve an evaluable parameter at the post-treatment follow-up.

## Conclusion

Rhinogenic headache is a well-represented clinical entity whose diagnosis can be easily made. The correct identification of the anatomical variants that cause the contact points’ presence allow us to recognize the specific trigger points.

The endoscopic surgical treatment is proposed as the pathology choice approach, considering the favorable results demonstrated both in the short–medium term and in the long term.

To identify the optimal treatment features of RCPH and in particular among the subgroups those most likely to surgical or medical treatment, future studies should describe in a precise and detailed manner the initial symptomatologic characteristics of the medical or surgical intervention. With these premises, it will be possible to directly compare the specific treatment outcomes in the short–medium and the long-term already in the study design.
